# Prognostic significance of AKR1C4 and the advantage of combining EBV DNA to stratify patients at high risk of locoregional recurrence of nasopharyngeal carcinoma

**DOI:** 10.1186/s12885-022-09924-3

**Published:** 2022-08-11

**Authors:** Shan-Shan Guo, Yan-Zhou Chen, Li-Ting Liu, Rong-Ping Liu, Yu-Jing Liang, Dong-Xiang Wen, Jing Jin, Lin-Quan Tang, Hai-Qiang Mai, Qiu-Yan Chen

**Affiliations:** 1 State Key Laboratory of Oncology in South China, Collaborative Innovation Center for Cancer Medicine, Guangdong Key Laboratory of Nasopharyngeal Carcinoma Diagnosis and Therapy, Sun Yat-Sen University Cancer Center, Guangzhou, China; 2 Department of Nasopharyngeal Carcinoma, Sun Yat-Sen University Cancer Center, Guangzhou, China

**Keywords:** AKR1C4, EBV DNA, Recurrence, Nomogram, Nasopharyngeal carcinoma

## Abstract

**Background:**

Distinguishing patients at a greater risk of recurrence is essential for treating locoregional advanced nasopharyngeal carcinoma (NPC). This study aimed to explore the potential of aldo–keto reductase 1C4 (AKR1C4) in stratifying patients at high risk of locoregional relapse.

**Methods:**

A total of 179 patients with locoregionally advanced NPC were grouped by different strategies; they were: (a) divided into two groups according to AKR1C4 expression level, and (b) classified into three clusters by integrating AKR1C4 and Epstein-Barr virus (EBV) DNA. The Kaplan–Meier method was used to calculate locoregional relapse-free survival (LRFS), overall survival (OS), progression-free survival (PFS), and distant metastasis-free survival (DMFS). The Cox proportional hazards model was used to determine potential prognostic factors, and a nomogram was generated to predict 3-year and 5-year LRFS.

**Results:**

A significant difference in the 5-year LRFS was observed between the high and low AKR1C4 expression groups (83.3% vs. 92.7%, respectively; *p* = 0.009). After integrating AKR1C4 expression and EBV DNA, the LRFS (84.7%, 84.5%, 96.9%, *p* = 0.014) of high-, intermediate-, and low- AKR1C4 and EBV DNA was also significant. Multivariate analysis indicated that AKR1C4 expression (*p* = 0.006) was an independent prognostic factor for LRFS. The prognostic factors incorporated into the nomogram were AKR1C4 expression, T stage, and EBV DNA, and the concordance index of the nomogram for locoregional relapse was 0.718.

**Conclusions:**

In conclusion, high AKR1C4 expression was associated with a high possibility of relapse in NPC patients, and integrating EBV DNA and AKR1C4 can stratify high-risk patients with locoregional recurrence.

**Supplementary Information:**

The online version contains supplementary material available at 10.1186/s12885-022-09924-3.

## Background

Nasopharyngeal carcinoma (NPC) is an epithelial-derived carcinoma with an unbalanced distribution worldwide. NPC is most prominent in southeast Asia, especially Southern China [1], contributing to more than 70% of new cases [2]. Intensity modulated radiotherapy (IMRT) is the main therapeutic method for non-metastatic NPC [3], and concomitant chemoradiotherapy with or without adjuvant or induction chemotherapy is primarily recommended for the treatment of locoregionally advanced NPC, according to NCCN guidelines [4]. Although current regimens for non-metastatic cases are considered to be effective due to the relatively optimistic 5-year overall survival rate, recurrence and distant metastasis, which directly lead to treatment failure, remain intractable for oncologists. The mechanisms for the development of recurrence and distant metastasis of NPC, albeit not fully been illustrated, had progresses these decades. For example, it had been found that spindle cells had great importance in predicting invasive/metastatic NPC due to its epithelial-mesenchymal transition and stem-like features [5, 6]. For patients with recurrent NPC, the distant metastasis rate is considerably higher than that for patients who are newly treated, and reirradiation shows low survival benefit and extensive risk of severe late toxicity [7, 8]. Hence, finding more biomarkers to recognize and classify patients with high recurrence risk before radical treatment enables oncologists to design more individualized and precise treatment plans.

Aldo–keto reductase (AKR) 1C4 is a member of the AKR superfamily, which consists of 14 families and over 150 enzymes. AKRs are widely expressed in prokaryotes and eukaryotes and are essential enzymes for the detoxification of aldehydes and ketones. AKR superfamily members can be categorized as aldose reductase, acetaldehyde reductase, hydroxysteroid dehydrogenase, or dihydrodiol dehydrogenase [9, 10]. Apart from the biochemical catalyzing process in cells, these enzymes also participate in oncological events such as cell protection, tumor generation, and cancer diagnosis [11]. For instance, AKR1B/1C families facilitate uterine tumors by influencing prostaglandin, estrogen, progesterone, and retinoid metabolism, stimulating cell proliferation and reducing cell differentiation [12]. In mammary and prostate carcinomas, AKR1C3 interacts with the steroid and prostaglandin pathways and promotes tumor progression [13, 14]. The expression of AKR1C2 also increases both non-small cell lung cancer and prostate cancer [15, 16]. As an important member of the AKR1C family, AKR1C4 expression was found to be elevated in colorectal carcinoma and lung cancer; however, there is no known study illustrating the prognostic value of AKR1C4 in NPC patients [17, 18]. Thus, we hypothesized that there may be a relationship between AKR1C4 and poor prognosis in NPC. In the present study, we examined the expression of AKR1C4 in newly diagnosed, locally advanced NPC patients who received radical irradiation-based therapy and sought clinical outcomes through statistical analysis. Our aim was to determine the prognosis-predicting efficacy of AKR1C4 and potential to guide individual treatment for NPC patients.

## Methods

### Ethical statement

This study was reviewed and approved by the Institutional Review Board of the Sun Yat-sen University Cancer Center (SYSUCC; Guangzhou, Guangdong, China). All research operations on patients were conducted in accordance with the Declaration of Helsinki, and written informed consent was obtained from all patients before the start of the study.

### Patients and follow-up

All tissue specimens for immunohistochemical (IHC) examination of AKR1C4 were obtained from 179 newly diagnosed NPC patients treated at SYSUCC, Guangzhou, China. All samples were collected after the diagnosis of NPC and prior to anti-cancer treatment. All patients following these criteria were retrospectively enrolled: (a) pathologically diagnosed, stage I-IVa (8^th^ edition of the AJCC/UICC staging system); (b) WHO type II-III; (c) enrolled between January 2010 and November 2011; (d) treated with IMRT alone or concurrent chemoradiotherapy or induction chemotherapy (PF; cisplatin and 5-fluorouracil [5-FU]-based) following concurrent (cisplatin-based) chemoradiotherapy; (e) available data of quantitative pretreatment EBV DNA load; and (f) no simultaneous severe heart, lung, liver, kidney diseases, or other cancers. EBV DNA was examined in the laboratory of the Department of Molecular Diagnosis at SYSUCC by quantitative real-time PCR as previously described [19, 20]. For induction chemotherapy, the regimen was PF, which included administration of cisplatin 80 mg/m^2^ intravenously (iv.) on day 1, and 5-FU 800 mg/m^2^ iv. on days 1–5 for 2 to 3 cycles every 3 weeks. The regimen for concurrent chemoradiotherapy was either 80–100 mg/m^2^ cisplatin iv. every 3 weeks. Monthly follow-ups were performed for the first 3 months after treatment completion, every 3 months for the following 3 years, every 6 months for the next 2 years, and annually thereafter. The median duration between follow-ups was 90 months (range: 4–119).

### Establishment of HONE1-IR cells and cell culture

HONE1 cells were exposed to 0–10 Gy X-rays (dose rate: 300 cGy/min) to confirm its sublethal dose. Then, the HONE1 cells were irradiated once with sublethal dose, and survived cells were cultured to be the first generation of subline cells. The subline cells were cultured and irradiated again with sublethal dose, and survived cells were cultured as the second generation of subline. Above-mentioned process was repeated for 5 times, generating radioresistant cells (HONE1-IR). HONE1, HONE1-IR cells and other NPC cell lines (HNE1, 6-10B, S18, S26, CNE1, CNE2, 5-8F, and SUNE2) were cultured in RPMI 1640 medium (Gibco) with 5% fetal bovine serum (FBS; Gibco). The nasopharyngeal epithelial cell line (NP69) was grown in keratinocyte serum-free medium supplemented with epidermal growth factor (EGF) (Invitrogen). All the cell lines were cultured in a humidified 5% CO_2_ incubator at 37 °C. All the cell lines had been authenticated and were generously provided by Prof. Mu-Sheng Zeng and Prof. Chao-Nan Qian (Sun Yat-sen University Cancer Center, Guangzhou, China).

### RNA sequencing of cell lines

HONE1 and HONE1-IR cells were lysed in TRIzol reagent (Invitrogen, Carlsbad, CA, USA), and the RNeasy kit (Qiagen, Hilden, Germany) was used to extract total RNA. The quality and quantitation of the RNA was examined by 2100 Bioanalyzer (Agilent) and NanoDrop 2000. The RNA-Seq libraries were performed using TruSeq® RNA Library Prep Kit v2 (Illumina, San Diego, CA, USA). Paired-end sequencing (2 × 150 bp) of the libraries was performed in HiSeq 2500 (Illumina). Raw reads were filtered to rule out low-quality data prior to analysis. Fragments Per Kilobase Per Million Mapped Fragments (FPKM) values were calculated to estimate the expression level of genes in different cell lines. Differentially expressed genes were deemed as false discovery rate ≤ 0.05 and fold change ≥ 2, and were visualized as heat map and scatter plot.

### Immunohistochemistry

To examine AKR1C4 expression in paraffin-embedded tissue samples, specific antibodies (AKR1C4 antibody, rabbit, DF9190; Affinity Biosciences, OH, USA; 1:100 dilution) were used and internally validated by the manufacturer. Paraffin sections were baked in oven at 60 °C for 2 h and were deparaffinized with xylene for 10 min twice and were subsequently rehydrated with graded ethanol. We then used 3% hydrogen peroxide to eliminate the influence of endogenous peroxidase, and 0.01 mmol/L citrate buffer (pH 6.0) was used for antigen retrieval in a high-pressure cooker. The incubation with primary antibodies was then conducted at 4 °C for 12 h. After being washed with PBS for three times, the specimens were subsequently incubated with Dako REAL EnVision/HRP Rabbit/Mouse reagent at a concentration of 1:100 for 30 min at 37 °C. The antigens were visualized using 3,3′-Diaminobenzidine substrates and the slides were counterstained with hematoxylin. Hydrochloric acid alcohol and lithium carbonate were used for the differentiation and bluing of the slices, respectively. An Olympus CCD camera, a Nikon Eclipse Ti-S microscope (× 400 magnification), and NIS-Elements F3.2 software were used to record staining results.

### Immunoblotting

Cells were collected and lysed in sodium dodecyl sulfate (SDS) sample buffer (62.5 mM Tris–HCl [pH 6.8], 3% SDS, 10% glycerol, 50 mM DL-dithiothreitol, and 0.1% bromophenol blue) containing protease inhibitors (Roche). BCA method (Pierce, Thermo Fisher Scientific, Rockford, IL, USA) was performed to determine protein concentration. Proteins (20 μg) were separated on sodium dodecyl sulfate–polyacrylamide gels and transferred to polyvinylidene difluoride membranes. The membranes were blocked by 5% bovine serum albumin in TBST (1 M Tris–HCl [pH = 7.5], 0.8% NaCl, and 0.1% Tween-20), incubated with primary antibody against AKR1C4 (cat no. GTX89330, Genetex) at 37 °C for 3 h, and incubated with horseradish peroxidase (HRP)-conjugated secondary antibody (cat no. #L3042, SAB). Enhanced chemiluminescence (Pierce, Rockford, IL, USA) was performed to visualize proteins.

### Scoring of immunohistochemistry and grouping strategies

AKR1C4 expression was evaluated by combining the intensity and positive rates. The staining intensity was classified as 0 to 3 (0 = negative; 1 = mild staining; 2 = moderate staining; 3 = strong staining), and the positive percentage was scored as follows: 1 point (stained cells ≤ 25%), 2 points (25% < stained cells ≤ 50%), and 3 points (stained cells > 50%). The total score was calculated by multiplying the intensity and positive percentage scores [21, 22]. Two independent pathologists blinded to the origin of the samples evaluated the IHC specimens and the scoring values were accepted if they reported consistent results. The expression status of AKR1C4 was divided into two groups based on the median of total score: low AKR1C4 expression (≤ 4) and high AKR1C4 expression (> 4). To examine whether AKR1C4 can increase the predictive efficacy of the widely used biomarker EBV DNA, we also integrated AKR1C4 expression and EBV DNA together and assigned patients into three clusters: (a) high-AKR1C4 and EBV DNA (AKR1C4 total score > 4 and EBV DNA level ≥ 4000 copies/ml); (b) low-AKR1C4 and EBV DNA (AKR1C4 total score ≤ 4 and EBV DNA level < 4000 copies/ml); or (c) intermediate-AKR1C4 and EBV DNA (other circumstances). The high and low EBV DNA levels were set to ≥ 4000 copies/ml and < 4000 copies/ml, respectively, based on previous studies that confirmed its strong prognostic value [23, 24].

### Statistical analyses

Comparisons of categorical variables of different AKR1C4 expression groups were analyzed using Chi-square tests or Fisher’s exact tests. The Mann–Whitney U test was used to compare the continuous variables. The Kaplan–Meier method and log-rank tests were used to analyze and compare the survival rates. Receiver operating characteristic (ROC) curves were generated and area under the curve (AUC) was used to compare the predictive efficacy of AKR1C4 level, EBV DNA level, and combined-AKR1C4 and EBV DNA level for LRFS. Locoregional recurrence-free survival (LRFS), progression-free survival (PFS), overall survival (OS), and distant metastasis-free survival (DMFS) were defined as the time interval from the start of treatment to the date of the advent of local or regional relapse, disease progression (death, recurrence, or distant metastasis), death from any cause, and observed distant lesion, respectively. Multivariate analysis was performed using the Cox proportional hazards model, and the results were calculated as hazard ratios (HR) and 95% confidence intervals (CIs). Body mass index (BMI), UICC clinical stage, T stage, N stage, EBV DNA, smoking history, and AKR1C4 expression were considered possible prognostic factors in this model.

A nomogram was generated based on the results of the multivariate analysis to visualize risk prediction. The nomogram was established to reflect the risks of locoregional relapse at 3 and 5 years. The concordance index (C-index) was calculated by comparing the survival probability of the nomogram predictions and Kaplan–Meier estimates to assess the predictive accuracy of the nomogram. As the C-index approaches 1.0, the nomogram-generated probabilities more closely match the true probabilities. Calibration of the nomogram for 5-year LRFS was performed using a calibration curve incorporating the model-predicted and actual observed probabilities. The more the calibration curve overlaps with the diagonal, the more accurate the nomogram-predicted probabilities are in accordance with the true probabilities.

Statistical analyses were performed using the Statistical Package for Social Sciences version 25.0 and GraphPad Prism version 8.4.0. The nomogram was formulated using the *rms* package of R, ver. 4.1.1. *P*-values < 0.05 were considered to be statistically significant (2-sided).

## Results

### Patient characteristics and IHC analysis

Among the 179 patients, 141 (79%) were male and 38 (21%) were female, with a median age of 50 years (range: 19–79). Five (2.8%) patients were diagnosed as stage I, 22 (12.3%) as stage II, 80 (44.7%) as stage III, and 72 (40.2%) as stage IVa. Most patients were pathologically diagnosed as WHO type III (96.6%). All patients were treated with IMRT-based radiotherapy and underwent concurrent chemoradiotherapy (cisplatin-based), induction chemotherapy (cisplatin and 5-FU-based), or radiotherapy alone.

**Fig. 1:**
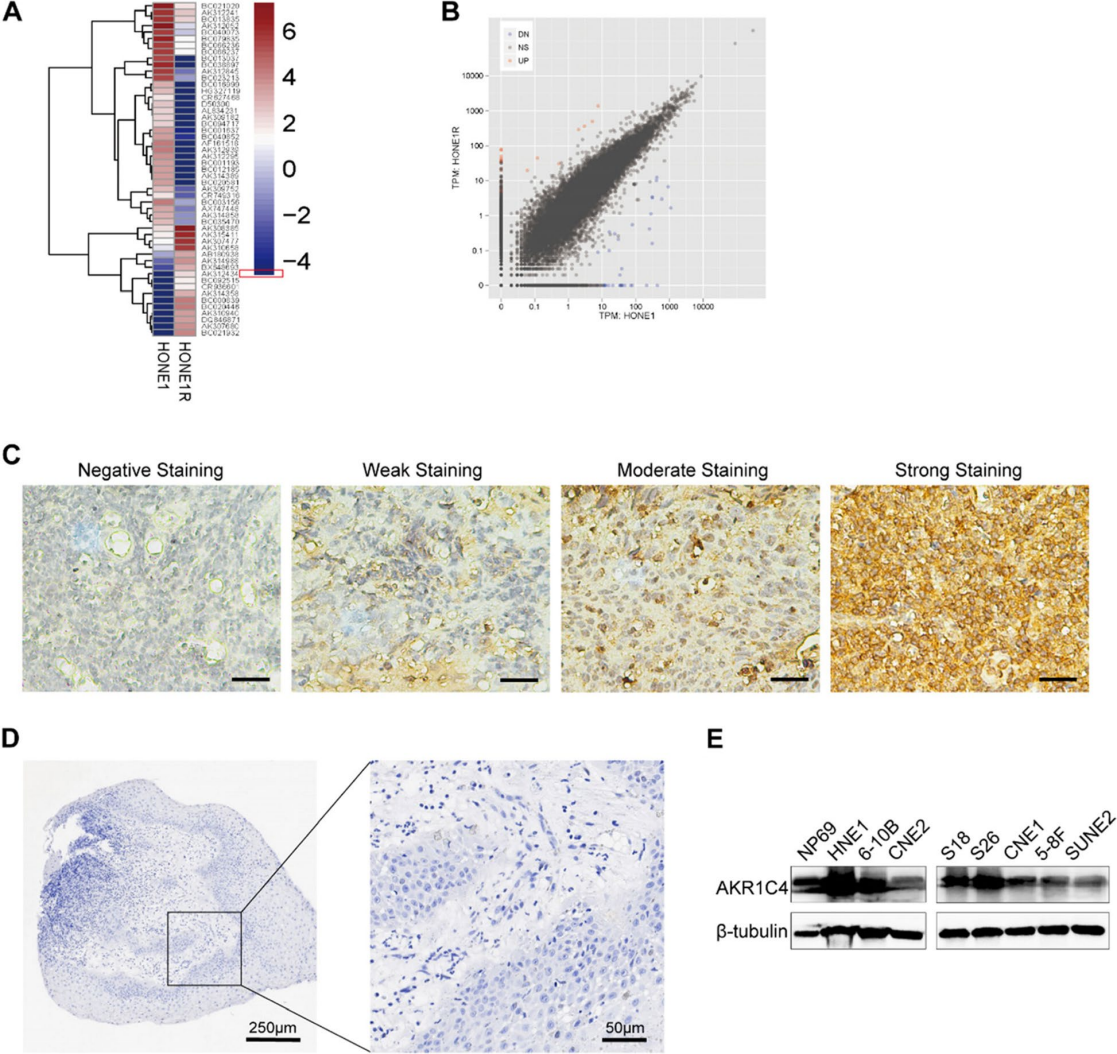
AKR1C4 expression profile. **A** Heat map demonstrated expression pattern of different genes in HONE1 vs. HONE1-IR. High or low expression was reflected as red or blue indicated in the scale bar, respectively. Herein, locus AK314988 (highlighted by red frame), which was AKR1C4, showed a significant higher expression in HONE1-IR than in HONE1. **B** In scatter plot, red and blue dots represented upregulation and downregulation of gene expression, respectively, and AKR1C4 expression was elevated in HONE1-IR cell line, which indicated a robust replicability of RNA-seq samples. **C** Representative images of different intensities of immunohistochemical staining for AKR1C4 in nasopharyngeal carcinoma (NPC) tissues (200 ×): Negative staining, weak staining, moderate staining, and strong staining. All micrographs were taken and processed at identical conditions. Scale bar: 50 μm. **D** IHC result of normal nasopharyngeal mucosa stained for AKR1C4, in magnification 40 × (left) and 200 × (right). **E** Protein levels of AKR1C4 in different NPC cell lines (HNE1, 6-10B, CNE2, S18, S26, CNE1, 5-8F and SUNE2) and normal nasopharyngeal epithelial cell line NP69 were evaluated by western blotting. β-tubulin was used as internal control

Radioresistant NPC cells (HONE1-IR) were established through sublethal doses of irradiation, and significant elevation of AKR1C4 expression was observed in HONE1-IR compared to that in HONE1 by RNA sequencing (Fig. 1A, 1B). We confirmed the AKR1C4 expression status in all 179 patients via IHC. AKR1C4 was positively expressed in nearly all patients (178/179, 99.4%) in terms of intensity. Representative images of different AKR1C4 expression intensities in NPC tissues are shown in Fig. 1C. IHC was also performed on normal nasopharyngeal mucosa, and the expression of AKR1C4 was negative. Meanwhile, different extent of expression of AKR1C4 was widely seen in NPC cell lines (HNE1, 6-10B, CNE2, S18, S26, CNE1, 5-8F and SUNE2) and in nasopharyngeal epithelial cell lines NP69 by western blotting. (Fig. 1D, 1E) Based on the total AKR1C4 score, the patients were divided into two groups: 105 patients (58.7%) had low AKR1C4 expression, and 74 patients (41.3%) had high AKR1C4 expression. Table 1 shows the baseline characteristics of the high and low AKR1C4 expression groups. The clinical stage was found to be significantly different between the two groups, with more advanced-stage (III or IVa) patients in the high AKR1C4 expression group (89.2% vs. 81.9%, respectively; *p* = 0.014). Other clinicopathological features were not significantly different between the two groups.

**Table 1:** Baseline demographic and clinical characteristics

**Characteristic**	**Low AKR1C4 expression (*n* = 105)**	**High AKR1C4 expression (*n* = 74)**	***P*** ^a^
Age			0.970
Median (Range)	50 (20–79)	49.5(19–78)	
Sex			0.914
Male	83 (79.0%)	58 (78.4%)	
Female	22 (21.0%)	16 (21.6%)	
T stage^b^			0.175
1	5 (4.8%)	6 (8.1%)	
2	21 (20.0%)	7 (9.5%)	
3	46 (43.8%)	40 (54.1%)	
4	33 (31.4%)	21 (28.4%)	
N stage^b^			0.847
0	24 (22.9%)	16 (21.6%)	
1	32 (30.5%)	23 (31.1%)	
2	32 (30.5%)	26 (35.1%)	
3	17 (16.2%)	9 (12.2%)	
Disease stage^b^			**0.014**
I	1 (1.0%)	4 (5.4%)	
II	18 (17.1%)	4 (5.4%)	
III IVa	41 (39.0%) 45 (42.9%)	39 (52.7%) 27 (36.5%)	
WHO type II III	4 (3.8%) 101 (96.2%)	2 (2.7%) 72 (97.3%)	1.000
EBV DNA (copies/mL) < 4000 ≥ 4000	67 (63.8%) 38 (36.2%)	41 (55.4%) 33 (44.6%)	0.258
VCA-IgA < 1:80 ≥ 1:80	32 (30.5%) 73 (69.5%)	20 (27.0%) 54 (73.0%)	0.617
EA-IgA			0.509
< 1:10	42 (40.0%)	26 (35.1%)	
≥ 1:10	63 (60.0%)	48 (64.9%)	
CRP (g/ml)			0.150
< 3.00	72 (68.6%)	43 (58.1%)	
≥ 3.00	33 (31.4%)	31 (41.9%)	
BMI (kg/m^2^)			0.228
< 18.5	11 (10.5%)	4 (5.4%)	
≥ 18.5	94 (89.5%)	70 (94.6%)	
Smoking status			0.972
Yes	40 (38.1%)	28 (37.8%)	
No	65 (61.9%)	46 (62.2%)	

*Abbreviations*: *BMI* Body mass index, *CRP* C-reactive protein, *EA* Early antigen, *EBV DNA* Epstein-Barr virus deoxyribonucleic acid, *VCA* Viral capsid antigen, *WHO* World Health Organization

^a^Boldface letter: significant

^b^According to the 8th edition of the American Joint Committee on Cancer/Union for International Cancer Control staging system

### AKR1C4 expression was an independent prognostic factor for LRFS

**Fig. 2:**
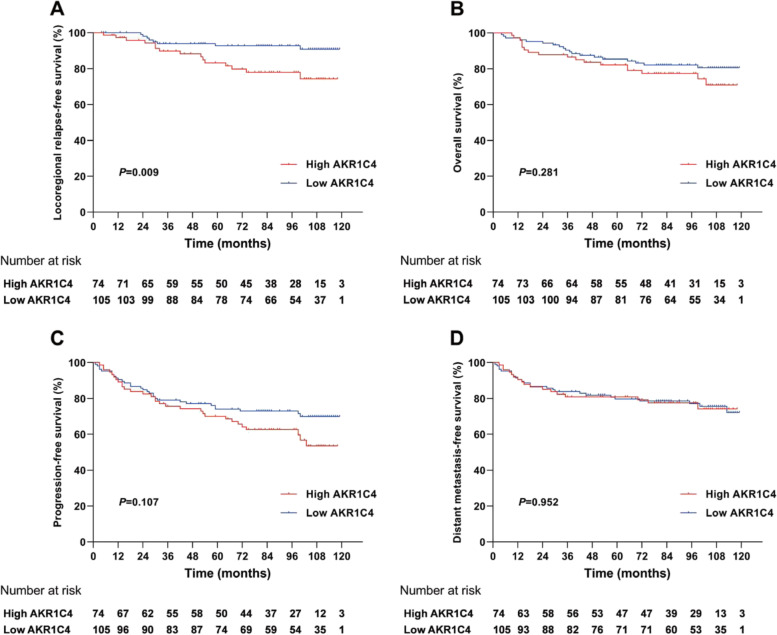
Kaplan–Meier curves of survival outcomes for patients between high (> 4) and low (≤ 4) AKR1C4 expression. **A** Locoregional relapse-free survival. **B** Overall survival. **C** Progression-free survival. **D** Distant metastasis-free survival

The median duration between follow-ups was 90 months (range: 4–119 months). The Kaplan–Meier method was used to compare the survival difference between the high and low AKR1C4 expression groups. No significant differences were observed between the two groups in OS, PFS, and DMFS rates (Fig. 2). However, the high AKR1C4 expression group was found to have a significantly lower 5-year LRFS rate than that of the low AKR1C4 expression group (83.3% vs. 92.7%, HR = 2.957, 95% CI = 1.278–6.841, *p* = 0.009) (Fig. 2).

The Cox proportional hazards model was used to identify the independent prognostic factors for LRFS, OS, PFS, and DMFS (Table 2). High expression of AKR1C4 was an independent prognostic factor for LRFS (*p* = 0.006, HR = 3.670, 95% CI = 1.462–9.215). EBV DNA level, T stage, and N stage were significant prognostic factors for OS (*p* = 0.032, 0.030, and 0.002; HR = 2.194, 3.477, and 3.548; 95% CI = 1.068–4.508, 1.127–10.729, and 1.593–7.900, respectively). There were four significant prognostic factors affecting PFS, which were EBV DNA (*p* = 0.004, HR = 2.282, 95% CI = 1.293–4.030), T stage (*p* = 0.027, HR = 2.680, 95% CI = 1.120–6.408), N stage (*p* = 0.006, HR = 2.302, 95% CI = 1.268–4.180), and smoking history (*p* = 0.039, HR = 1.718, 95% CI = 1.027–2.875). The significant prognostic factors for DMFS were EBV DNA (*p* = 0.002, HR = 3.012, 95% CI = 1.478–6.139) and N stage (*p* = 0.002, HR = 3.541, 95% CI = 1.598–7.845).

**Table 2:** Multivariable analysis of prognostic factors for nasopharyngeal carcinoma patients (AKR1C4 included)

**Endpoint**	**Factor**	**HR (95% CI)**	***P*** ^a^
**LRFS**			
	AKR1C4 expression (high vs. low)	3.670 (1.462–9.215)	**0.006**
	T stage (4 vs. 1/2/3)	4.970 (0.600–41.201)	0.137
	N stage (2/3 vs. 0/1)	1.157 (0.465–2.878)	0.754
	Disease stage (IVa vs. I/II/III)	0.477 (0.057–4.016)	0.496
	EBV DNA (≥ 4000 vs. < 4000) copies/ml	1.834 (0.741–4.539)	0.190
	BMI (≥ 18.5 vs. < 18.5 kg/m^2^)	0.411 (0.087–1.942)	0.262
	Smoking history (yes vs. no)	2.024 (0.877–4.670)	0.098
**OS**			
	AKR1C4 expression (high vs. low)	1.605 (0.803–3.207)	0.181
	T stage (4 vs. 1/2/3)	3.477 (1.127–10.729)	**0.030**
	N stage (2/3 vs. 0/1)	3.548 (1.593–7.900)	**0.002**
	Disease stage (IVa vs. I/II/III)	0.744 (0.232–2.386)	0.619
	EBV DNA (≥ 4000 vs. < 4000) copies/ml	2.194 (1.068–4.508)	**0.032**
	BMI (≥ 18.5 vs. < 18.5 kg/m^2^)	0.385 (0.148–1.007)	0.052
	Smoking history (yes vs. no)	1.616 (0.832–3.138)	0.157
**PFS**			
	AKR1C4 expression (high vs. low)	1.576 (0.918–2.705)	0.099
	T stage (4 vs. 1/2/3)	2.680 (1.120–6.408)	**0.027**
	N stage (2/3 vs. 0/1)	2.302 (1.268–4.180)	**0.006**
	Disease stage (IVa vs. I/II/III)	0.918 (0.377–2.234)	0.850
	EBV DNA (≥ 4000 vs. < 4000) copies/ml	2.282 (1.293–4.030)	**0.004**
	BMI (≥ 18.5 vs. < 18.5 kg/m^2^)	0.537 (0.230–1.254)	0.151
	Smoking history (yes vs. no)	1.718 (1.027–2.875)	**0.039**
**DMFS**			
	AKR1C4 expression (high vs. low)	1.045 (0.539–2.026)	0.897
	T stage (4 vs. 1/2/3)	2.272 (0.915–5.642)	0.077
	N stage (2/3 vs. 0/1)	3.541 (1.598–7.845)	**0.002**
	Disease stage (IVa vs. I/II/III)	1.193 (0.462–3.084)	0.715
	EBV DNA (≥ 4000 vs. < 4000) copies/ml	3.012 (1.478–6.139)	**0.002**
	BMI (≥ 18.5 vs. < 18.5 kg/m^2^)	0.458 (0.190–1.105)	0.082
	Smoking history (yes vs. no)	1.619 (0.873–3.002)	0.126

*Abbreviations*: *BMI* Body mass index, *CI* Confidence interval, *DMFS* Distant metastasis-free survival, *EBV DNA* Epstein-Barr virus deoxyribonucleic acid, *HR* hazard ratio, *LRFS* Locoregional relapse-free survival, *OS* overall survival, *PFS* progression-free survival

^a^Boldface letter: significant

### Integrating AKR1C4 and EBV DNA to stratify patients from different risks

**Fig. 3:**
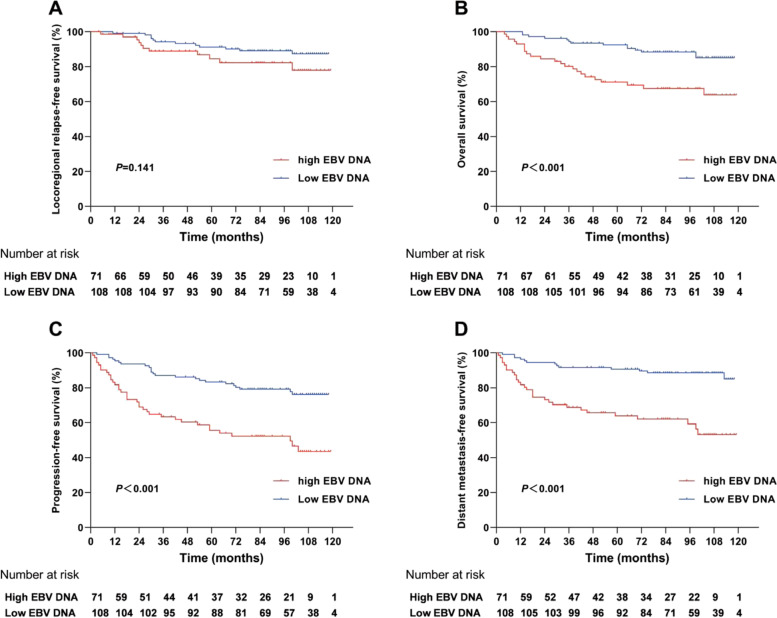
Kaplan–Meier curves of survival outcomes for patients between high EBV DNA level (≥ 4000 copies/ml) and low EBV DNA level (< 4000 copies/ml) patients. **A** Locoregional relapse-free survival. **B** Overall survival. **C** Progression-free survival. **D** Distant metastasis-free survival

**Fig. 4:**
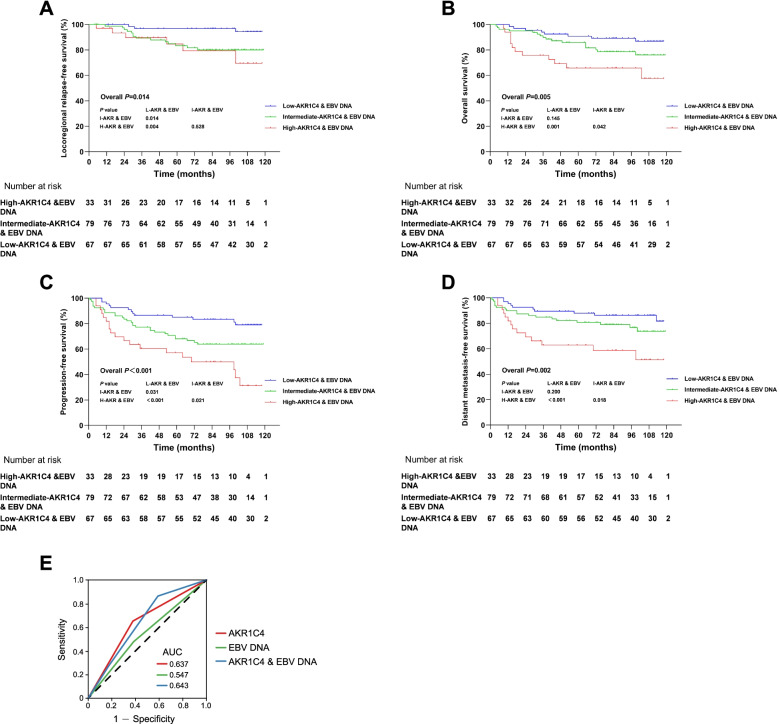
Kaplan–Meier curves of survival outcomes for patients between high-AKR1C4 and EBV DNA, intermediate-AKR1C4 and EBV DNA, and low-AKR1C4 and EBV DNA patients. Patients with high AKR1C4 (> 4) and high EBV DNA level (≥ 4000 copies/ml) simultaneously were defined as high-AKR1C4 and EBV DNA, low AKR1C4 (≤ 4) and low EBV DNA level (< 4000 copies/ml) were grouped as low-AKR1C4 and EBV DNA, and the rest of the circumstances were considered as intermediate-AKR1C4 and EBV DNA, which were abbreviated as H-AKR & EBV, L-AKR & EBV, and I-AKR & EBV, respectively. **A** Locoregional relapse-free survival. **B** Overall survival. **C** Progression-free survival. **D** Distant metastasis-free survival. **E** Receiver operating characteristic (ROC) curve of three different models predicting recurrence of NPC patients. Area under the curve (AUC) indicated the predictive efficacy, the closer the AUC reaches 1, the higher the efficacy predicting recurrence of the model is. The AUC of survival model consisted of AKR1C4 and EBV DNA has higher AUC than model constructed by AKR1C4 or EBV DNA alone

We also conducted survival analysis for LRFS, OS, PFS, and DMFS, using EBV DNA as a prognostic factor. The survival curves showed opposite results compared to AKR1C4 (Fig. 3), and the OS, PFS, and DMFS rates were significantly lower in the high EBV DNA group than those in the low EBV DNA group (*p* < 0.001), whereas LRFS showed no significance. We combined EBV DNA and AKR1C4 expression levels as a new variable to stratify patients into three different groups. The integrated index showed that the high-AKR1C4 and EBV DNA group had worse survival outcomes than the intermediate- and low-AKR1C4 and EBV DNA groups, all statistically significant in 5-year LRFS (84.7%, 84.5%, 96.9%, *p* = 0.014), OS (65.7%, 87.0%, 90.8%, *p* = 0.005), PFS (57.1%, 69.0%, 85.0%, *p* < 0.001), and DMFS (62.9%, 81.8%, 88.0%, *p* = 0.002) in Kaplan–Meier analysis, indicating a universal prognostic applicability (Fig. 4A-4D). Moreover, with regard to OS, PFS, and DMFS, significant differences were found between the high-AKR1C4 and EBV DNA group and either the intermediate- or low-AKR1C4 and EBV DNA groups. In contrast, significant differences were only observed between the low-AKR1C4 and EBV DNA group and both intermediate- and high-AKR1C4 and EBV DNA groups in LRFS, whereas the intermediate- vs. high-AKR1C4 and EBV DNA groups showed no significant differences. ROC analysis was also performed to evaluate the effect of AKR1C4 level, EBV DNA level, and the combined level of AKR1C4 and EBV DNA on recurrence. The AUC was 0.637, 0.547, and 0.643, respectively. (Fig. 4E).

Multivariable analysis was also performed (Table 3). Similar to previous results, AKR1C4 and EBV DNA were the sole significant prognostic factors for LRFS (high-AKR1C4 and EBV DNA vs. low-AKR1C4 and EBV DNA, *p* = 0.007, HR = 7.696, 95% CI = 1.736–33.248; intermediate- vs. low-AKR1C4 and EBV DNA, *p* = 0.011, HR = 5.182, 95% CI = 1.451–18.505). For OS, the significant factors were AKR1C4 and EBV DNA (high- vs. low-AKR1C4 and EBV DNA, *p* = 0.009, HR = 3.567, 95% CI = 1.380–9.224), T stage (*p* = 0.031, HR = 3.425, 95% CI = 1.121–10.471), N stage (*p* = 0.001, HR = 3.863, 95% CI = 1.717–8.693), and BMI (*p* = 0.045, HR = 0.378, 95% CI = 0.146–0.979). Significant factors for PFS were AKR1C4 and EBV DNA (high vs. low, *p* = 0.001, HR = 3.601, 95% CI = 1.688–7.684; intermediate vs. low, *p* = 0.016, HR = 2.310, 95% CI = 1.172–4.552), T stage (*p* = 0.031, HR = 2.588, 95% CI = 1.091–6.139), and N stage (*p* = 0.003, HR = 2.520, 95% CI = 1.379–4.603). The significant prognostic factors for DMFS were AKR1C4 and EBV DNA (high vs. low, *p* = 0.014, HR = 2.993, 95% CI = 1.248–7.176), N stage (*p* = 0.001, HR = 4.009, 95% CI = 1.798–8.937), and BMI (*p* = 0.032, HR = 0.383, 95% CI = 0.159–0.919).

**Table 3:** Multivariable analysis of prognostic factors for nasopharyngeal carcinoma patients (combination of AKR1C4 and EBV DNA included)

**Endpoint**	**Factor**	**HR (95% CI)**	***P*** ^a^
**LRFS**			
	AKR1C4 + EBV DNA		
	Intermediate risk vs. low	5.182 (1.451–18.505)	**0.011**
	High risk vs. low	7.596 (1.736–33.248)	**0.007**
	T stage (4 vs. 1/2/3)	6.102 (0.740–50.285)	0.093
	N stage (2/3 vs. 0/1)	1.245 (0.498–3.114)	0.640
	Disease stage (IVa vs. I/II/III)	0.377 (0.046–3.097)	0.364
	BMI (≥ 18.5 vs. < 18.5 kg/m^2^)	0.465 (0.099–2.181)	0.331
	Smoking history (yes vs. no)	1.752 (0.746–4.114)	0.198
**OS**			
	AKR1C4 + EBV DNA		
	Intermediate risk vs. low	2.345 (0.985–5.581)	0.054
	High risk vs. low	3.567 (1.380–9.224)	**0.009**
	T stage (4 vs. 1/2/3)	3.425 (1.121–10.471)	**0.031**
	N stage (2/3 vs. 0/1)	3.863 (1.717–8.693)	**0.001**
	Disease stage (IVa vs. I/II/III)	0.781 (0.248–2.461)	0.673
	BMI (≥ 18.5 vs. < 18.5 kg/m^2^)	0.378 (0.146–0.979)	**0.045**
	Smoking history (yes vs. no)	1.577 (0.806–3.806)	0.183
**PFS**			
	AKR1C4 + EBV DNA		
	Intermediate risk vs. low	2.310 (1.172–4.552)	**0.016**
	High risk vs. low	3.601 (1.688–7.684)	**0.001**
	T stage (4 vs. 1/2/3)	2.588 (1.091–4.603)	**0.031**
	N stage (2/3 vs. 0/1)	2.520 (1.379–4.603)	**0.003**
	Disease stage (IVa vs. I/II/III)	0.976 (0.407–2.340)	0.956
	BMI (≥ 18.5 vs. < 18.5 kg/m^2^)	0.518 (0.223–1.201)	0.125
	Smoking history (yes vs. no)	1.679 (0.996–2.830)	0.052
**DMFS**			
	AKR1C4 + EBV DNA		
	Intermediate risk vs. low	1.835 (0.820–4.106)	0.140
	High risk vs. low	2.993 (1.248–7.176)	**0.014**
	T stage (4 vs. 1/2/3)	1.851 (0.752–4.559)	0.181
	N stage (2/3 vs. 0/1)	4.009 (1.798–8.937)	**0.001**
	Disease stage (IVa vs. I/II/III)	1.456 (0.569–3.727)	0.433
	BMI (≥ 18.5 vs. < 18.5 kg/m^2^)	0.383 (0.159–0.919)	**0.032**
	Smoking history (yes vs. no)	1.655 (0.886–3.091)	0.114

*Abbreviations*: *BMI* Body mass index *CI* Confidence interval, *DMFS* Distant metastasis-free survival, *EBV DNA* Epstein-Barr virus deoxyribonucleic acid, *HR* Hazard ratio, *LRFS* Locoregional relapse-free survival, *OS* Overall survival, *PFS* Progression-free survival

^a^Boldface letter: significant

### Developing a nomogram for recurrence using AKR1C4 expression

**Fig. 5:**
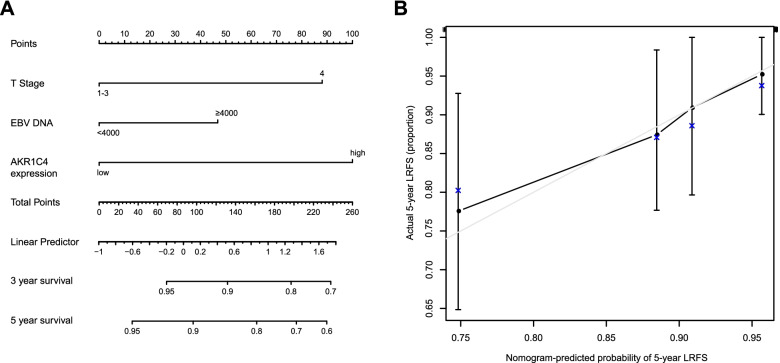
Nomogram predicting 3-year and 5-year survival in NPC patients. **A** The nomogram consists of T stage, EBV DNA, and AKR1C4 level. **B** Calibration curve for predicting local recurrence at 5 years. The actual 5-year LRFS is plotted on the Y-axis, and the nomogram-predicted probability of local recurrence is plotted on the X-axis. EBV DNA, Epstein-Barr virus deoxyribonucleic acid

In Fig. 5A, we present a nomogram that incorporates the AKR1C4 expression level, T stage, and EBV DNA. The values for each variable correspond to points on the scale axis, ranging from 0 to 100. After summing up all points, this total value must be located on the “total points” axis. Next, a vertical line should be drawn from the “total points” axis to the survival axes, and the probabilities of 3-year recurrence and 5-year recurrence can be determined. It can be inferred from the nomogram that AKR1C4 affects LRFS the most, followed by T stage, EBV DNA, and N stage. The C-index of the nomogram was 0.718, indicating that it could be a good model for predicting recurrence. The calibration curve showed a marked overlap with the diagonal, suggesting a strong predictive ability. (Fig. 5B).

## Discussion

Traditionally, EBV DNA is regarded as a robust prognostic indicator in NPC patients [2]. However, besides EBV DNA, there may be other predictive biomarkers, especially for specific aspects such as recurrence or distant metastasis. Research shows that radioresistance plays an essential role in treatment failure, especially in remnant disease and locoregional relapse after radical radiotherapy [25, 26]. To the best of our knowledge, this is the first study to focus on the prognostic value of AKR1C4 in NPC using IHC to intuitively visualize AKR1C4 expression, and this is the first study to integrate AKR1C4 into a nomogram that predicts tumor relapse.

AKR1C4 had a wide expression in NPC, both in tissue specimens and in NPC cells, and it was barely expressed in normal nasopharyngeal mucosa. The reliability of AKR1C4 as biomarker associated with NPC has thus been proved. Moreover, we found that AKR1C4 was expressed more in advanced-stage (III or IVa) patients than in early-stage patients, which may be attributed to its ability to promote tumorigenesis and progression. In our Kaplan–Meier analysis, high expression of AKR1C4 was not associated with worse OS, PFS, and DMFS, but was significantly associated with LRFS. In the low AKR1C4 group, recurrence rarely occurred after 5 years, but the OS rate continued to decrease, possibly due to non-cancer deaths. This difference might have caused the insignificant OS rate between the two groups. In the multivariate analysis, AKR1C4 expression was also the sole significant prognostic factor for LRFS after adjusting for factors such as tumor stage, BMI, and smoking history. In a ten-year analysis of NPC patients treated with definitive IMRT, T category and age were prognostic factors for recurrence [27]. It should be noted that when AKR1C4 was added to the survival analysis, the association between AKR1C4 and LRFS was prominent enough to cover the influence of T stage, suggesting that AKR1C4 alone was sufficient to discriminate patients with high recurrence risk. Apart from traditionally used EBV DNA, there are other non-invasive biomarkers that are associated with poor prognosis of NPC, such as miR-214-3p, pretreatment serum lactate dehydrogenase, Ki-67, and radiomic features [28–31]. The lack of long-term follow-up time, ease of being influenced by body conditions such as inflammation, and complexity of the algorithm and analysis hindered their clinical application. AKR1C4 and its testing method can overcome the aforementioned drawbacks. Survival analysis revealed strong correlations between EBV DNA and OS, PFS, and DMFS. Therefore, AKR1C4 and EBV DNA could possibly have a complementary role in predicting prognosis, which was not provided by either biomarker alone. The results of Kaplan–Meier and multivariable analyses demonstrated a significant association between integrated AKR1C4 and EBV DNA and all endpoints, suggesting a more favorable ability to distinguish patients from different risks. It is worth mentioning that patients with high- or intermediate-AKR1C4 and EBV DNA still had local relapse more so than low-AKR1C4 and EBV DNA patients, which was consistent with results using AKR1C4 only. Therefore, integrating AKR1C4 and EBV DNA together improved the prognostic value.

To visualize the recurrence probability, we established a nomogram based on the results of Cox regression analysis. According to previous publications, there are various factors that contribute to NPC recurrence; for instance, plasma EBV DNA, T stage, age, gender, and invasion extent [32–35]. T stage, especially T4, had a poorer LRFS rate than T1–T3 [27]. Ethmoid invasion and gross tumor volume were also risk factors for LRFS, reflecting the extent of invasion [35]. Thus, T stage was included in the nomogram, and its influence on LRFS was second only to that of AKR1C4 expression. This nomogram provided a visual prediction of local recurrence, and had favorable consistency with clinical data based on the C-index and calibration curve. It may be convenient for oncologists to utilize this nomogram to screen NPC patients with a high recurrence risk in clinical practice. Generally, AKR1C4 has the potential to guide risk stratification and treatment individualization, such as using a more intense regimen in patients with high recurrence risk.

Studies show that radioresistance is a major cause of tumor relapse [36]. Our study clarified the relationship between AKR1C4 and recurrence in NPC, and also indicated an assosiation between radioresistance and recurrence in NPC. The mechanism of radioresistance in NPC remains unclear, and studies elucidating how AKR1C4 promotes radioresistance remain scarce. Some related genes, molecules, and signal pathways have been found to contribute to radioresistance, such as the STAT1/IFN, cyclin D1/DNA-PK/AKT/GSK3β, and ATM/Chk2/p53 pathways, and miRNA-95, CLIC4, and CHK1/2 [37–42]. Other AKR family members, such as AKR1C3 [43] enhances radioresistance of prostate cancer cells via the MAPK pathway. Xie et al.[44] revealed the role of AKR1C3 in IL-6-mediated radioresistance in NSCLC. Xiong et al.[45] showed that AKR1C3 overexpression modulates oxidative stress and increases resistance to radiation in esophageal carcinomas. Thus, AKR1C4 may share similar mechanism of radioresistance. Despite the scarcity of radioresistance-related literature on AKR1C4, AKR1C4 was found to be correlated with drug resistance through metabolic inactivation by carbonyl reduction[46], and that resistance could be reversed by mefenamic acid [47]. Drug resistance mediated by AKR1C4 demonstrates that because of the natural enzymatic role of AKR, it is likely that AKR1C4 catalyzes specific biochemical reactions and regulates tumor metabolism and oxidative stress, or uses non-metabolic routes such as gene repair and epigenetic modification to facilitate radioresistance in NPC. More laboratory studies exploring the mechanisms of AKR1C4 and its recurrence are warranted, and using AKRs as therapeutic targets also has a promising future [48].

Our study had some limitations. The first limitation is the retrospective nature of this study. Although the baseline characteristics were comparable and the long follow-up duration made the results more convincing, inevitable selection bias may still exist. Second, this was a single center study, and all patients were enrolled in an endemic area where most of the pathological types were WHO type III. To expand our results to other regions of the world, a well-designed, multicenter study is required. Third, the nomogram we developed lacked external validation; therefore, further verification in different patient cohorts is urgently required to guarantee its clinical applicability.

## Conclusions

In conclusion, our study uncovered an association between AKR1C4 expression and recurrence in NPC, and integrating EBV DNA and AKR1C4 stratified high-risk patients with locoregional recurrence. A nomogram combining AKR1C4 and other factors showed potential for predicting recurrence in NPC patients.

## Supplementary Information

**Additional file 1**.

## Data Availability

The datasets generated and/or analyzed during the current study are available in the Research Data Deposit public platform (www.researchdata.org.cn), with the approval RDD number as RDDB2022311425.

## Abbreviations

AJCC: American Joint Committee on Cancer
AKR1C4: Aldo–keto reductase 1C4
BMI: Body mass index
CIs: Confidence intervals
CRP: C-reactive protein
DMFS: Distant metastasis-free survival
EA: Early antigen
EBV: Epstein-Barr virus
HR: Hazard ratio
IHC: Immunohistochemistry
IMRT: Intensity modulated radiotherapy
LRFS: Locoregional recurrence-free survival
NCCN: National Comprehensive Cancer Network
NPC: Nasopharyngeal carcinoma
OS: Overall survival
PBS: Phosphate buffered saline
PFS: Progression-free survival
UICC: Union for International Cancer Control
VCA: Viral capsid antigen
WHO: World Health Organization

## References

[1] Tang LL, Chen WQ, Xue WQ, He YQ, Zheng RS, Zeng YX, Jia WH (2016). Global trends in incidence and mortality of nasopharyngeal carcinoma. Cancer Lett.

[2] Chen YP, Chan ATC, Le QT, Blanchard P, Sun Y, Ma J (2019). Nasopharyngeal carcinoma. Lancet.

[3] Peng G, Wang T, Yang KY, Zhang S, Zhang T, Li Q, Han J, Wu G (2012). A prospective, randomized study comparing outcomes and toxicities of intensity-modulated radiotherapy vs. conventional two-dimensional radiotherapy for the treatment of nasopharyngeal carcinoma. Radiother Oncol.

[4] Colevas AD, Yom SS, Pfister DG, Spencer  S, Adelstein D, Adkins D, Brizel DM, Burtness B, Busse PM, Caudell JJ (2018). NCCN Guidelines Insights: Head and Neck Cancers, Version 1.2018.. J Natl Compr Canc Netw.

[5] Luo W, Yao K (2013). Molecular characterization and clinical implications of spindle cells in nasopharyngeal carcinoma: a novel molecule-morphology model of tumor progression proposed. PLoS ONE.

[6] Luo WR, Chen XY, Li SY, Wu AB, Yao KT (2012). Neoplastic spindle cells in nasopharyngeal carcinoma show features of epithelial-mesenchymal transition. Histopathology.

[7] Lee AW, Law SC, Foo W, Poon YF, Cheung FK, Chan DK, Tung SY, Thaw M, Ho JH (1993). Retrospective analysis of patients with nasopharyngeal carcinoma treated during 1976–1985: survival after local recurrence. Int J Radiat Oncol Biol Phys.

[8] Liu LT, Chen QY, Tang LQ, Zhang L, Guo SS, Guo L, Mo HY, Zhao C, Guo X, Chen MY (2016). With or without reirradiation in advanced local recurrent nasopharyngeal carcinoma: a case-control study. BMC Cancer.

[9] Ellis EM (2007). Reactive carbonyls and oxidative stress: potential for therapeutic intervention. Pharmacol Ther.

[10] Jez JM, Flynn TG, Penning TM (1997). A new nomenclature for the aldo-keto reductase superfamily. Biochem Pharmacol.

[11] Zhang S, Wen B, Zhou B, Yang L, Cha C, Xu S, Qiu X, Wang Q, Sun H, Lou X (2013). Quantitative analysis of the human AKR family members in cancer cell lines using the mTRAQ/MRM approach. J Proteome Res.

[12] Rizner TL (2012). Enzymes of the AKR1B and AKR1C subfamilies and uterine diseases. Front Pharmacol.

[13] Penning TM, Steckelbroeck S, Bauman DR, Miller MW, Jin Y, Peehl DM, Fung KM, Lin HK (2006). Aldo-keto reductase (AKR) 1C3: role in prostate disease and the development of specific inhibitors. Mol Cell Endocrinol.

[14] Penning TM, Burczynski ME, Jez JM, Hung CF, Lin HK, Ma H, Moore M, Palackal N, Ratnam K (2000). Human 3alpha-hydroxysteroid dehydrogenase isoforms (AKR1C1-AKR1C4) of the aldo-keto reductase superfamily: functional plasticity and tissue distribution reveals roles in the inactivation and formation of male and female sex hormones. Biochem J.

[15] Wang HW, Lin CP, Chiu JH, Chow KC, Kuo KT, Lin CS, Wang LS (2007). Reversal of inflammation-associated dihydrodiol dehydrogenases (AKR1C1 and AKR1C2) overexpression and drug resistance in nonsmall cell lung cancer cells by wogonin and chrysin. Int J Cancer.

[16] Rizner TL, Lin HK, Peehl DM, Steckelbroeck S, Bauman DR, Penning TM (2003). Human type 3 3alpha-hydroxysteroid dehydrogenase (aldo-keto reductase 1C2) and androgen metabolism in prostate cells. Endocrinology.

[17] Gylfe AE, Katainen R, Kondelin J, Tanskanen T, Cajuso T, Hänninen U, Taipale J, Taipale M, Renkonen-Sinisalo L, Järvinen H (2013). Eleven candidate susceptibility genes for common familial colorectal cancer. PLoS Genet.

[18] Ter-Minassian M, Asomaning K, Zhao Y, Chen F, Su L, Carmella SG, Lin X, Hecht SS, Christiani DC (2012). Genetic variability in the metabolism of the tobacco-specific nitrosamine 4-(methylnitrosamino)-1-(3-pyridyl)-1-butanone (NNK) to 4-(methylnitrosamino)-1-(3-pyridyl)-1-butanol (NNAL). Int J Cancer.

[19] Shao JY, Li YH, Gao HY, Wu QL, Cui NJ, Zhang L, Cheng G, Hu LF, Ernberg I, Zeng YX (2004). Comparison of plasma Epstein-Barr virus (EBV) DNA levels and serum EBV immunoglobulin A/virus capsid antigen antibody titers in patients with nasopharyngeal carcinoma. Cancer.

[20] Zhang LL, Huang MY, Fei X, Wang KX, Song D, Wang T, Sun LY, Shao JY (2020). Risk stratification for nasopharyngeal carcinoma: a real-world study based on locoregional extension patterns and Epstein-Barr virus DNA load. Ther Adv Med Oncol.

[21] Waisberg J, De Souza VL, Affonso Junior RJ, Silva SR, Denadai MV, Margeotto FB, De Souza CS, Matos D (2014). Overexpression of the ITGAV gene is associated with progression and spread of colorectal cancer. Anticancer Res.

[22] Huang PY, Guo SS, Zhang Y, Lu JB, Chen QY, Tang LQ, Zhang L, Liu LT, Zhang L, Mai HQ (2016). Tumor CTLA-4 overexpression predicts poor survival in patients with nasopharyngeal carcinoma. Oncotarget.

[23] Chan AT, Lo YM, Zee B, Chan LY, Ma BB, Leung SF, Mo F, Lai M, Ho S, Huang DP (2002). Plasma Epstein-Barr virus DNA and residual disease after radiotherapy for undifferentiated nasopharyngeal carcinoma. J Natl Cancer Inst.

[24] Leung SF, Zee B, Ma BB, Hui EP, Mo F, Lai M, Chan KC, Chan LY, Kwan WH, Lo YM (2006). Plasma Epstein-Barr viral deoxyribonucleic acid quantitation complements tumor-node-metastasis staging prognostication in nasopharyngeal carcinoma. J Clin Oncol.

[25] Baskar R, Yap SP, Chua KL, Itahana K (2012). The diverse and complex roles of radiation on cancer treatment: therapeutic target and genome maintenance. Am J Cancer Res.

[26] Lee AWM, Ng WT, Chan JYW, Corry J, Mäkitie A, Mendenhall WM, Rinaldo A, Rodrigo JP, Saba NF, Strojan P (2019). Management of locally recurrent nasopharyngeal carcinoma. Cancer Treat Rev.

[27] Wu LR, Liu YT, Jiang N, Fan YX, Wen J, Huang SF, Guo WJ, Bian XH, Wang FJ, Li F (2017). Ten-year survival outcomes for patients with nasopharyngeal carcinoma receiving intensity-modulated radiotherapy: an analysis of 614 patients from a single center. Oral Oncol.

[28] Wang J, Xu Y, Wang J, Ying H (2020). Circulating miR-214-3p predicts nasopharyngeal carcinoma recurrence or metastasis. Clin Chim Acta.

[29] Long G, Tang W, Fu X, Liu D, Zhang L, Hu G, Hu G, Sun W (2019). Pre-treatment serum lactate dehydrogenase predicts distant metastasis and poor survival in nasopharyngeal carcinoma. J Cancer.

[30] Zhao Y, Shen L, Huang X, Jing D, Huang D, Fu J, Li Z, Zhang G, Shen L (2017). High expression of Ki-67 acts a poor prognosis indicator in locally advanced nasopharyngeal carcinoma. Biochem Biophys Res Commun.

[31] Zhang LL, Huang MY, Li Y, Liang JH, Gao TS, Deng B, Yao JJ, Lin L, Chen FP, Huang XD (2019). Pretreatment MRI radiomics analysis allows for reliable prediction of local recurrence in non-metastatic T4 nasopharyngeal carcinoma. EBioMedicine.

[32] Wang WY, Twu CW, Lin WY, Jiang RS, Liang KL, Chen KW, Wu CT, Shih YT, Lin JC (2011). Plasma Epstein-Barr virus DNA screening followed by ^18^F-fluoro-2-deoxy-D-glucose positron emission tomography in detecting posttreatment failures of nasopharyngeal carcinoma. Cancer.

[33] Hong RL, Lin CY, Ting LL, Ko JY, Hsu MM (2004). Comparison of clinical and molecular surveillance in patients with advanced nasopharyngeal carcinoma after primary therapy: the potential role of quantitative analysis of circulating Epstein-Barr virus DNA. Cancer.

[34] Tang LQ, Li CF, Li J, Chen WH, Chen QY, Yuan LX, Lai XP, He Y, Xu YX, Hu DP et al: Establishment and validation of prognostic nomograms for endemic nasopharyngeal carcinoma. J Natl Cancer Inst 2016, 108(1). 10.1093/jnci/djv291 26467665

[35] Zhang LL, Li YY, Hu J, Zhou GQ, Chen L, Li WF, Lin AH, Ma J, Qi ZY, Sun Y (2018). Proposal of a pretreatment nomogram for predicting local recurrence after intensity-modulated radiation therapy in T4 nasopharyngeal carcinoma: a retrospective review of 415 Chinese patients. Cancer Res Treat.

[36] Kong L, Wang L, Shen C, Hu C, Wang L, Lu JJ (2016). Salvage intensity-modulated radiation therapy (IMRT) for locally recurrent nasopharyngeal cancer after definitive IMRT: a novel scenario of the modern era. Sci Rep.

[37] Khodarev NN, Beckett M, Labay E, Darga T, Roizman B, Weichselbaum RR (2004). STAT1 is overexpressed in tumors selected for radioresistance and confers protection from radiation in transduced sensitive cells. Proc Natl Acad Sci U S A.

[38] Shimura T, Kakuda S, Ochiai Y, Nakagawa H, Kuwahara Y, Takai Y, Kobayashi J, Komatsu K, Fukumoto M (2010). Acquired radioresistance of human tumor cells by DNA-PK/AKT/GSK3beta-mediated cyclin D1 overexpression. Oncogene.

[39] Huang X, Taeb S, Jahangiri S, Emmenegger U, Tran E, Bruce J, Mesci A, Korpela E, Vesprini D, Wong CS (2013). miRNA-95 mediates radioresistance in tumors by targeting the sphingolipid phosphatase SGPP1. Cancer Res.

[40] Nie X, Guo E, Wu C, Liu D, Sun W, Zhang L, Long G, Mei Q, Wu K, Xiong H (2019). SALL4 induces radioresistance in nasopharyngeal carcinoma via the ATM/Chk2/p53 pathway. Cancer Med.

[41] Zhu L, Chen Q, Zhang L, Hu S, Zheng W, Wang C, Bai Y, Pan Y, Konishi T, Guan J (2020). CLIC4 regulates radioresistance of nasopharyngeal carcinoma by iNOS after γ-rays but not carbon ions irradiation. Am J Cancer Res.

[42] Wang WJ, Wu SP, Liu JB, Shi YS, Huang X, Zhang QB, Yao KT (2013). MYC regulation of CHK1 and CHK2 promotes radioresistance in a stem cell-like population of nasopharyngeal carcinoma cells. Cancer Res.

[43] Sun SQ, Gu X, Gao XS, Li Y, Yu H, Xiong W, Yu H, Wang W, Li Y, Teng Y (2016). Overexpression of AKR1C3 significantly enhances human prostate cancer cells resistance to radiation. Oncotarget.

[44] Xie L, Yu J, Guo W, Wei L, Liu Y, Wang X, Song X (2013). Aldo-keto reductase 1C3 may be a new radioresistance marker in non-small-cell lung cancer. Cancer Gene Ther.

[45] Xiong W, Zhao J, Yu H, Li X, Sun S, Li Y, Xia Q, Zhang C, He Q, Gao X (2014). Elevated expression of AKR1C3 increases resistance of cancer cells to ionizing radiation via modulation of oxidative stress. PLoS ONE.

[46] Wsol V, Szotakova B, Martin HJ, Maser E (2007). Aldo-keto reductases (AKR) from the AKR1C subfamily catalyze the carbonyl reduction of the novel anticancer drug oracin in man. Toxicology.

[47] Shiiba M, Yamagami H, Yamamoto A, Minakawa Y, Okamoto A, Kasamatsu A, Sakamoto Y, Uzawa K, Takiguchi Y, Tanzawa H (2017). Mefenamic acid enhances anticancer drug sensitivity via inhibition of aldo-keto reductase 1C enzyme activity. Oncol Rep.

[48] Zeng CM, Chang LL, Ying MD, Cao J, He QJ, Zhu H, Yang B (2017). Aldo-Keto reductase AKR1C1-AKR1C4: functions, regulation, and intervention for anti-cancer therapy. Front Pharmacol.

