# Supplementary figures and images for: Prognostic significance of AKR1C4 and the advantage of combining EBV DNA to stratify patients at high risk of locoregional recurrence of nasopharyngeal carcinoma
Source: BMC Cancer. 2022 Aug 11;22:880. doi: 10.1186/s12885-022-09924-3 (PMC9373296; doi:10.1186/s12885-022-09924-3)

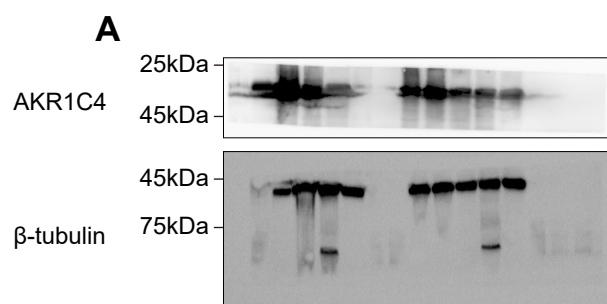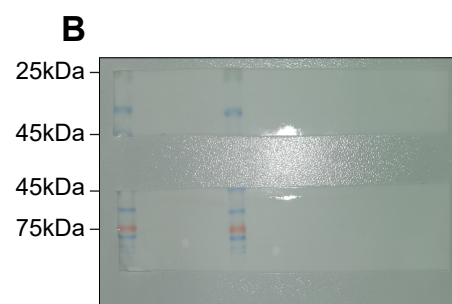

Supplement: Supplementary file 1 — Additional file 1. [file 12885_2022_9924_MOESM1_ESM.pdf]
